# Genetic and epigenetic alterations of Ras signalling pathway in colorectal neoplasia: analysis based on tumour clinicopathological features

**DOI:** 10.1038/sj.bjc.6604014

**Published:** 2007-10-09

**Authors:** K Harada, S Hiraoka, J Kato, J Horii, H Fujita, K Sakaguchi, Y Shiratori

**Affiliations:** 1 Department of Gastroenterology and Hepatology, Okayama University Graduate School of Medicine, Dentistry and Pharmaceutical Sciences, Okayama, Japan

**Keywords:** RASSF2, methylation, *K-ras/BRAF* mutations, Ras signalling pathway, colorectal adenoma, colorectal cancer

## Abstract

Activation of RAS signalling induced by *K-ras/BRAF* mutations is a hallmark of colorectal tumours. In addition, Ras association domain families 1 and 2 (RASSF1 and RASSF2), the negative regulators of *K-ras*, are often inactivated by methylation of the promoter region in those tumours. However, reports showing differences in the occurrence of these alterations on the basis of tumour characteristics have been scarce. We analysed *K-ras/BRAF* mutations and the methylation status of RASSF1 and RASSF2 promoter regions in 120 colorectal adenomas with respect to their clinicopathological features. *K-ras/BRAF* mutations and RASSF2 methylation were observed in 49 (41%) and 30 (25%) of the samples, respectively, while RASSF1 methylation was observed in only 3 (2.5%). Adenomas with RASSF2 methylation often carried *K-ras/BRAF* mutations simultaneously (22 out of 30, *P*<0.01). Multivariate analysis revealed that the concomitance of these alterations was frequently observed in serrated adenomas (odds ratio (OR) 11.11; 95% confidence interval (CI) 1.96–63.00), but rarely in adenomas located in the sigmoid or descending colon (OR 0.13; 95% CI 0.03–0.58). A comparison between adenomas and cancers showed a significantly higher prevalence of these alterations in cancers than in adenomas in the proximal colon (58 *vs* 27%, *P*=0.02). Frequency and the time point of the occurrence of Ras signalling disorders differ according to colorectal neoplasia’s characteristics, particularly the location.

The Ras family of small guanosine triphosphatases plays essential roles in controlling the activity of several crucial signalling pathways (Malumbres and Barbacid, 2003). In normal cells, Ras proteins, including *H-*, *K-*, and *N-ras*, are transiently activated in response to extracellular signals and function as molecular switches in cell proliferation. Human tumours frequently express Ras proteins that have been activated by point mutation, although the spectrum of Ras gene mutation varies in different cancer types (Hidaka *et al*, 2000; Toyooka *et al*, 2006). *K-ras* mutation is frequently (∼45%) detected in colorectal tumours and has been thought to be an early event in colorectal tumorigenesis, mainly occurring during the transformation of small adenomas to intermediate-size ones (Fearon and Vogelstein, 1990; Kinzler and Vogelstein, 1996; Rajagopalan *et al*, 2002; Yuen *et al*, 2002). The activated guanosine triphosphate-bound Ras then interacts with several effector proteins, of which the Raf kinases are among the most well characterised (O’Neill *et al*, 2004). Raf is a major proliferative and antiapoptotic effector, and it was recently shown that *BRAF*, one of the Raf kinases, is frequently activated by mutation in human tumours, particularly melanomas (∼70%) and colorectal tumours (∼15%) (Davies *et al*, 2002; Rajagopalan *et al*, 2002; Yuen *et al*, 2002).

The recent discovery of the Ras association domain family (RASSF) protein of Ras effectors allows an explanation of at least some of the growth-inhibitory actions of Ras. To date, RASSF1–8 have been identified (Falvella *et al*, 2006), and RASSF1,2,4,5 have been shown to mediate Ras-dependent cell cycle arrest and apoptotic death (Vos *et al*, 2003). Moreover, these proteins are all frequently downregulated during tumour development by promoter CpG island methylation (Dammann *et al*, 2003; Vos *et al*, 2003; Aoyama *et al*, 2004; Eckfeld *et al*, 2004). Of these Ras effectors, RASSF1 and RASSF2 have been shown to be involved in colorectal tumorigenesis (van Engeland *et al*, 2002; Akino *et al*, 2005). Previous studies have found RASSF1 methylation in 20–45% of colorectal cancers (CRCs) (Wagner *et al*, 2002; van Engeland *et al*, 2002; Oliveira *et al*, 2005; Miranda *et al*, 2006). Meanwhile, Sakamoto *et al* (2004) have shown that the early flat-type colorectal tumours exhibited a much higher frequency of RASSF1 methylation (81.3%). The frequency of RASSF2 methylation in CRC was reported to be 42–73% (Akino *et al*, 2005; Hesson *et al*, 2005; Park *et al*, 2006).

It is generally accepted that a large proportion of CRCs develop from colorectal adenomas. The frequency of *K-ras* mutation in adenomas was found to be lower (3–17%) than that of CRCs, and the mutation had a strong association with larger adenoma size, villous histology, and high-grade dysplasia (Maltzman *et al*, 2001; Barry *et al*, 2006; Einspahr *et al*, 2006). In addition, our previous report indicated that the laterally spreading type of adenomas, particularly in the proximal colon, frequently carried the *K-ras* mutation (Hiraoka *et al*, 2006). The *BRAF* mutation was frequently observed in serrated adenomas and has been shown to be significantly correlated with the recently proposed serrated polyp-microsatellite instability pathway (Kambara *et al*, 2004). It has also been shown that the *K-ras* and *BRAF* mutations are mutually exclusive (Domingo *et al*, 2004; Li *et al*, 2006). On the other hand, reports regarding the frequency of RASSF1 and RASSF2 methylation in adenomas have been scarce. In addition, clinicopathological features of colorectal tumours that carry the methylation of either RASSF1 or RASSF2 are still largely unknown in adenomas as well as in cancers. Moreover, the association between *K-ras/BRAF* mutations and RASSF methylation, particularly whether or not they work synergistically or are mutually exclusive, is controversial in CRCs (Akino *et al*, 2005; Hesson *et al*, 2005; Park *et al*, 2006), and has not been sufficiently investigated in adenomas.

In this study, therefore, we investigated *K-ras* and *BRAF* mutations and the methylation status of RASSF1 and RASSF2 in colorectal adenoma samples. We then examined the correlation between these mutations and methylation based on the clinicopathological features of the adenomas. In particular, we focused on locational differences in combinations of these genetic and epigenetic alterations because a locational imbalance in each of these alterations has been indicated (Samowitz *et al*, 2000; Luchtenborg *et al*, 2005; Park *et al*, 2006). Moreover, we compared these alterations in adenomas with those in CRCs, and examined when these alterations are likely to occur during colorectal carcinogenesis.

## MATERIALS AND METHODS

### Patients and tissue samples

Tissues of adenomas larger than 10 mm were consecutively collected from patients who underwent endoscopic polypectomy or surgical resection of colorectal polyps at Okayama University Hospital between June 2003 and August 2005. Tissues from patients who had concurrent advanced CRC or a history of CRC were excluded from analysis. Also excluded were tissues from patients with inflammatory bowel disease or who had a known history of familial adenomatous polyposis or hereditary nonpolyposis CRC. A total of 97 patients met the criteria and provided written informed consent, and 120 adenomas from these patients were analysed. At the time of resection, patient's age and gender, and the location, size, and macroscopic appearance of each adenoma were determined. The anatomical distribution of adenoma locations divided among the caecum, ascending colon, transverse colon, descending colon, sigmoid colon, and rectum. In this study, locational grouping was divided into three categories: the proximal colon, including the caecum, ascending colon, and transverse colon; the distal colon, including the descending colon and sigmoid colon; and the rectum. Adenoma size was recorded as the maximum diameter of the extirpated specimen. The macroscopic appearance of the adenomas was classified as either the protruded type or the flat type. The flat type was defined as lesions with a low vertical axis extending laterally along the interior luminal wall, which is often called a laterally spreading tumour in Japan (Kudo *et al*, 1997). Adenomas forming protruded morphologies, other than the flat type, were designated as the protruded type.

In addition to the adenoma samples, 65 sporadic CRC tissue samples from patients who underwent surgical treatment at Okayama University Hospital were also collected and analysed.

A small tissue fragment was excised from resected neoplasm for DNA extraction, and the remaining portion was submitted for histological diagnosis. Samples were stored at −80°C until the analysis began.

This study protocol was approved by the Institutional Review Board of Okayama University Graduate School of Medicine, Dentistry and Pharmaceutical Sciences, and informed consent was obtained from each patient.

### Histopathological analysis of colorectal adenomas

Histologic studies were performed on all removed adenomas. The resected adenomas were fixed and embedded in paraffin. Serial sections were obtained and stained with haematoxylin and eosin. All cases were reviewed by two board certified pathologists, and were classified as tubular, tubulovillous, villous, or serrated adenomas. Hyperplastic polyps were not included in this analysis.

### Analysis of *K-ras* and *BRAF* mutations

To detect genetic alterations in *K-ras* and *BRAF*, we analysed the point mutations of codons 12 and 13 of the *K-ras* gene and the mutation of the exon 15 codon 600 of the *BRAF* gene by direct sequencing using a Big Dye Terminator v3.1, Cycle Sequencing kit, and an ABI Genetic Analyzer 3100. The primers for *K-ras* sequencing of codons 12 and 13 were similar to those described previously (Hiraoka *et al*, 2006). The primers for sequencing the *BRAF* gene were as follows: 5′-TCATAATGCTTGCTCTGATAGGA-3′ (forward) and 5′-TCCACTGATTAAATTTTTGGCC-3′ (reverse).

### Methylation analysis of RASSF1 and RASSF2

Tumour tissues were assayed for RASSF1 and RASSF2 promoter CpG island methylation using combined bisulphite restriction analysis (COBRA), following the report by Akino *et al* (2005). Extraction and bisulphite modification of genomic DNA from neoplastic tissues were performed as described previously (Hiraoka *et al*, 2006). Bisulphite-treated DNA was amplified by touchdown polymerase chain reaction (PCR) using primers capable of annealing both methylated and unmethylated alleles. Primer sequences for RASSF1 and RASSF2, and restriction enzymes that digest only methylated alleles were similar to those used in the previous report (Akino *et al*, 2005). A touchdown thermal cycle programme was modified in our analysis. In detail, that included an initial denaturation for 5 min at 95°C, followed by three cycles of 30 s at 95°C, 15 s at 63°C (RASSF1) or 67°C (RASSF2), and 30 s at 72°C. The annealing temperature was then decreased by 2°C until it reached 57°C (RASSF1) or 61°C (RASSF2): 61°C/65°C (4 cycles), 59°C/63°C (5 cycles), and 57°C/61°C (28 cycles), respectively, for RASSF1/RASSF2. A final extension for 4 min at 72°C was included at the end of the cycles before holding at 12°C. Samples digested with enzymes were electrophoresed on 6% polyacrylamide gels and visualised under ultraviolet light by staining with ethidium bromide.

### Statistical analysis

Differences in frequency were assessed by Fisher's exact test. Mann–Whitney *U*-test or Kruskal–Wallis rank test was used for comparisons of patient age. *P*-values less than 0.05 were considered significant. Multiple testing was corrected by Bonferroni correction, and *P*<0.017 is considered statistically significant in the comparison among three locational groups. Univariate and multivariate analyses were also performed to identify factors that were independently associated with the concomitance of *K-ras/BRAF* mutations and RASSF2 methylation, using a logistic regression model with corresponding calculation of odds ratios (ORs) and 95% confidence intervals (CIs). These analyses were performed using the SAS program (version 9.1, SAS Institute, Cary, NC, USA).

## RESULTS

### Clinicopathological characteristics of patients and adenomas

A total of 120 adenomas from 97 patients with a median age of 67 years (range 31–86) were analysed (Table 1). Of 97 patients, 79 had one adenoma, 14 had two adenomas, 3 had three adenomas, and 1 had four adenomas. Out of 14 patients harbouring two adenomas, 9 patients had both adenomas in the same location (two in the proximal colon, six in the distal colon, and one in the rectum). Three or four adenomas in the same patients did not cluster in the same location. Patients with more than one adenoma were significantly younger than those with a single adenoma (60 *vs* 67 years, *P*=0.02).

Of these 120 adenomas, 48 (40%) were located in the proximal colon (11 (9%) in the caecum, 24 (20%) in the ascending colon, and 13 (11%) in the transverse colon); 49 (41%) were in the distal colon (9 (8%) in the descending colon and 40 (33%) in the sigmoid colon); and 23 (19%) in the rectum. There were no significant differences in patient age among these three locational groups (*P*=0.31).

Histological examinations revealed that 72 (60%) were tubular adenomas, 40 (33%) were tubulovillous adenomas, and 8 (7%) were serrated adenomas.

### *K-ras/BRAF* mutations and RASSF1 and RASSF2 methylation

*K-ras* codons 12 and 13 and *BRAF* codon 600 point mutations of adenomas were analysed by direct sequencing analysis. Of the 120 adenomas, 49 (41%) exhibited *K-ras/BRAF* mutations. Of these, 43 carried the *K-ras* mutation and 6 carried the *BRAF* mutation, and there was no overlap between the two mutations. Mutations were significantly more likely to be observed in females (59%, *P*=0.01), adenomas larger than 2 cm (61%, *P*<0.01), and flat-type adenomas (59%, *P*<0.01) than in the respective counterparts. *K-ras* mutation was frequently detected in tubulovillous adenomas (24 out of 40, 60%), while *BRAF* mutation was mostly observed in serrated adenomas (5 out of 8, 63%). The analysis of locational distribution showed a higher prevalence of *K-ras/BRAF* mutations in the proximal colon (23 out of 48, 48%), and rectum (12 out of 23, 52%), and a lower prevalence in the distal colon (14 out of 49, 29%).

**Figure 1:**
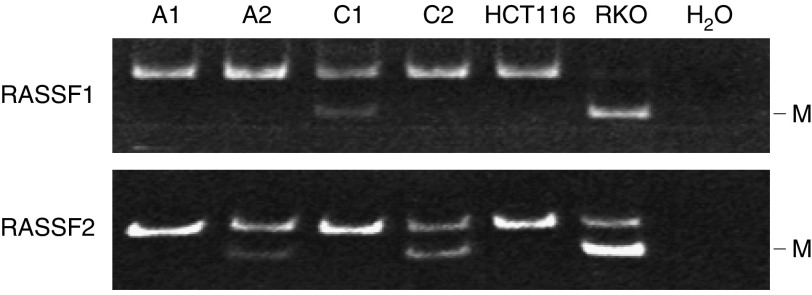
Analysis of methylation status of RASSF1 and RASSF2 promoter CpG island. COBRA was carried out using bisulphite-treated DNA from colorectal adenomas and cancers. A representative result for tumour tissue samples with/without RASSF1 or RASSF2 promoter methylation is shown. A1 is a sample without RASSF1 and RASSF2 methylation. A2 and C2 are samples with RASSF2 methylation, and without RASSF1 methylation. C1 is a sample with RASSF1 methylation, and without RASSF2 methylation. A, adenoma samples; C, cancer samples; M, methylated alleles; HCT116, colon cancer cell line used as a negative control for RASSF1 and RASSF2 methylation; RKO, colon cancer cell line used as a positive control for RASSF1 and RASSF2 methylation; H_2_O, sample without DNA.

Next, we analysed the methylation status of the promoter CpG islands of RASSF1 and RASSF2 (Figure 1). Of the 120 adenomas examined, RASSF2 methylation was observed in 30 (25%) cases, while only 3 (2.5%) adenomas exhibited RASSF1 methylation. In addition, 2 out of 3 adenomas with RASSF1 methylation also showed methylation of RASSF2. Therefore, subsequent analysis was performed on the basis of the results of RASSF2 methylation. RASSF2 methylation was significantly more frequently observed in large adenomas (⩾2 cm) than in adenomas of 1–2 cm (37 *vs* 19%, *P*=0.046). More strikingly, histopathological findings revealed that a large proportion of serrated adenomas (6 out of 8, 75%) exhibited RASSF2 methylation. The locational distribution of RASSF2 methylation was similar to that of *K-ras/BRAF* mutations, although the prevalence in each location was relatively low. Prevalence was higher in the proximal colon (14 out of 48, 29%) and rectum (9 out of 23, 39%), and lower in the distal colon (7 out of 49, 14%) (Table 1).

Adenomas with RASSF2 methylation were more likely to carry *K-ras/BRAF* mutations than those without RASSF2 methylation (73 *vs* 30%, *P*<0.01), suggesting that these genetic and epigenetic alterations are mutually correlated and may work synergistically. Patients who had at least one adenoma carrying either *K-ras/BRAF* mutations or RASSF2 methylation or both were significantly older than those who had adenoma(s) without these alterations (70 *vs* 62 years, *P*=0.01). In 18 patients harbouring plural adenomas, 2 patients each carried two adenomas with *K-ras/BRAF* mutations, while no patients had two or more adenomas with RASSF2 methylation. In addition, in only 4 out of 18 (22%) patients, all of adenomas that each patient carried exhibited the same *K-ras/BRAF* and RASSF2 status (all exhibited neither *K-ras/BRAF* mutation nor RASSF2 methylation). Thus, characteristics of adenomas in each patient were not always the same regarding *K-ras/BRAF* mutations or RASSF2 methylation.

### Uneven locational distribution of adenomas carrying *K-ras/BRAF* mutations and RASSF2 methylation

As described above, there was a locational imbalance among *K-ras/BRAF* mutations or RASSF2 methylation in adenomas. To elucidate the differences in the contribution of the Ras signalling pathway to neoplastic development according to colonic sites, we next investigated locational distribution of adenomas with either *K-ras/BRAF* mutations or RASSF2 methylation, or both (Table 1). Adenomas with either *K-ras/BRAF* mutations, RASSF2 methylation, or both were more frequently observed in the rectum than in the distal colon (65 *vs* 37%, *P*=0.04; and 26 *vs* 6%, *P*=0.03, respectively). Table 1 also shows that the prevalence of *K-ras/BRAF* mutations and/or RASSF2 methylation in the proximal colon was similar to that in the rectum (*K-ras/BRAF* mutations and RASSF2 methylation; 27% in the proximal colon *vs* 26% in the rectum, *P*>0.99), and thus the prevalence in the distal colon was significantly lower than that in the proximal colon (*K-ras/BRAF* mutations and RASSF2 methylation; 6 *vs* 27%, *P*<0.01). In other words, only the adenomas in the distal colon exhibited a distinctively lower prevalence of alterations of the Ras signalling pathway.

### Univariate and multivariate analyses of factors influencing concomitance of *K-ras/BRAF* mutations and RASSF2 methylation

Our results suggested that *K-ras/BRAF* mutations and RASSF2 methylation can cooperate and work synergistically in adenomas. In addition, there should be an imbalance in location or in other characteristics of adenomas that carry these alterations. Then, to identify factors for the concomitance of *K-ras/BRAF* mutations and RASSF2 methylation in adenomas, univariate and multivariate logistic regression analyses were performed with parameters including gender, age, location, morphology, size, and histological diagnosis (Table 2). In multivariate analysis, serrated adenoma was identified as a highly significant factor for the coexistence of *K-ras/BRAF* mutations and RASSF2 methylation (OR 11.11; 95% CI 1.96–63.00). In addition, location at the distal colon (the descending colon and sigmoid colon) was a significant factor for the absence of alterations in the Ras signalling pathway (OR 0.13; 95% CI 0.03–0.58). No other factors were proven to be associated with the coexistence of *K-ras/BRAF* mutations and RASSF2 methylation in the multivariate analysis.

### *K-ras/BRAF* mutations and RASSF2 methylation in CRC

**Figure 2:**
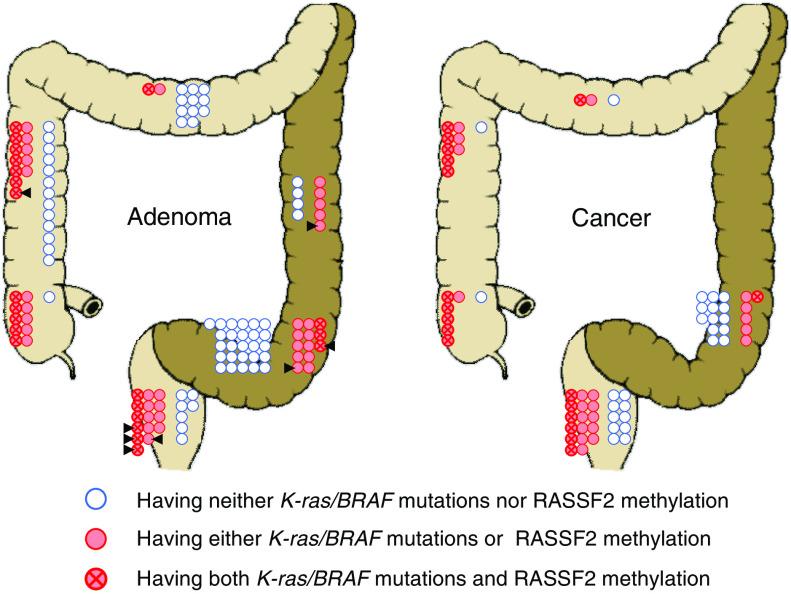
Locational distributions of adenomas and cancers with *K-ras/BRAF* mutation and/or RASSF2 methylation. Cancers with *K-ras/BRAF* mutation and/or RASSF2 methylation exhibit locational distribution similar to that of adenomas. However, the proportion of tumours in the proximal colon with both *K-ras/BRAF* mutation and RASSF2 methylation was significantly higher in cancers (58%) than in adenomas (27%) (*P*=0.02). Arrowheads indicate serrated adenomas.

Lastly, we analysed *K-ras/BRAF* mutations and RASSF2 methylation in CRC according to colonic site. Figure 2 compares the status of *K-ras/BRAF* mutations and RASSF2 methylation between adenomas and CRCs. Among 65 CRCs, the coexistence of *K-ras/BRAF* mutations and RASSF2 methylation was observed in 11 out of 19 (58%) lesions in the proximal colon, 1 out of 19 (5%) of those in the distal colon, and 6 out of 27 (22%) of those in the rectum. In the distal colon, adenomas and CRCs exhibited a similarly lower prevalence of disorders in the Ras signalling pathway. In addition, in the rectum, the proportions of tumours with both *K-ras/BRAF* mutations and RASSF2 methylation, with either of them, and with neither of them, were quite similar between adenomas and CRCs. In contrast, in the proximal colon, the proportion of tumours with both *K-ras/BRAF* mutations and RASSF2 methylation was significantly higher in CRCs (58%) than in adenomas (27%) (*P*=0.02). These results suggest that in the proximal colon, disorders of the Ras signalling pathway are almost requisite for development into carcinoma, while in the rectum two types of cancer development may exist: pathways associated with disorders of Ras signalling and those that are not. In the distal colon, the major route of CRC development does not seem to require disorders of the Ras signalling pathway.

## DISCUSSION

In the current study, we examined *K-ras/BRAF* mutations and methylation status of RASSF1 and RASSF2 in colorectal adenomas in relation to clinicopathological features. We also compared these genetic and epigenetic alterations in adenomas with those in cancers. Our results revealed that these genetic and epigenetic alterations were likely to occur concomitantly in each colorectal tumour. Furthermore, tumours with these alterations showed uneven locational distribution in the colorectum, and the distributions were a little different between adenomas and cancers.

Previous reports have shown differences between the right-side colon (including the caecum, ascending colon, and transverse colon) and the left-side colon (including the descending colon, sigmoid colon, and rectum) in epidemiologic incidence (Gonzalez *et al*, 2001), morphology (Okamoto *et al*, 2005), and molecular alterations (Iacopetta, 2002; Azzoni *et al*, 2007). Meanwhile, other reports revealed these differences between the colon and rectum (Frattini *et al*, 2004; Kim *et al*, 2006). Also regarding *K-ras/BRAF* mutations, several reports have shown a higher prevalence of *K-ras* mutation in the rectum than in the colon (Luchtenborg *et al*, 2005; Barry *et al*, 2006; Einspahr *et al*, 2006), while other reports compared *K-ras* mutation between the proximal colon and the distal colon, and found dominance in the former (Samowitz *et al*, 2000; Miranda *et al*, 2006). *BRAF* mutation is also known to be associated with the proximal localisation (Li *et al*, 2006). Our results seem consistent with these previous reports. Moreover, our results indicated that *K-ras/BRAF* mutations in adenomas were significantly less frequent in the distal colon than in either the proximal colon or the rectum. This suggests that *K-ras/BRAF* mutations are less important to neoplasia in the distal colon, although neoplasm occurs in greater numbers in the distal colon than in the other locations.

Meanwhile, only a few reports have referred to the correlation between clinicopathological features of colorectal neoplasia and methylation status of RASSF genes. Particularly scarce are reports regarding this issue in adenomas. We found that RASSF2 methylation was, like *K-ras/BRAF* mutations, frequently observed in large adenomas and in serrated adenomas. Moreover, the locational distribution of adenomas with this epigenetic alteration was also similar to that of adenomas with *K-ras/BRAF* mutations, although the frequency of the epigenetic change as a whole was lower than that of the genetic changes. As a result, genetic and epigenetic alterations in the Ras signalling pathway are likely to coexist and may work synergistically even in adenomas. These cooperative alterations were frequently observed in adenomas in the proximal colon and the rectum, while, as the multivariate analysis showed, those alterations were relatively rare in adenomas in the distal colon. These findings suggest that an alternative carcinogenic pathway other than the Ras signalling pathway may function in the distal colon.

In this study, we examined the methylation status of RASSF1 and RASSF2, because the methylation of these two genes among the RASSF series was reported to be specifically correlated with CRC development (van Engeland *et al*, 2002; Hesson *et al*, 2005). However, methylation of RASSF1 in colorectal neoplasm was relatively infrequent in our study (3% in adenomas and 12% (data not shown) in CRCs). Previous reports showed a higher prevalence of RASSF1 methylation in CRC (20–45%) (van Engeland *et al*, 2002; Wagner *et al*, 2002; Oliveira *et al*, 2005; Miranda *et al*, 2006). Furthermore, Sakamoto *et al* (2004) reported that 81.3% of flat-type tumours exhibited RASSF1 methylation. However, all of these previous reports used methylation-specific PCR (MSP) or its modified version as the methodology for detecting methylation. Since MSP has been shown to be too sensitive, resulting in overestimation of methylation status, the positive results of MSP are not always consistent with the loss of function of the gene. In contrast, we used COBRA, a stricter methodology than MSP, following the method of Akino *et al* (2005) (Hiraoka *et al*, 2006). COBRA for RASSF1 has been proven to be correlated with its gene function (Akino *et al*, 2005), and our results were close to theirs (13% in CRCs and 6% in adenomas). In this regard, RASSF1 methylation would be less important than has been considered thus far.

In the case of RASSF2, there is a problem similar to that with RASSF1 regarding the interpretation of experimental results. The majority of reports adopted MSP as the experimental procedure, and reported a higher prevalence of methylation (70–73% in CRCs and 88–100% in adenomas) (Hesson *et al*, 2005; Park *et al*, 2006) than in our study (46% in CRCs and 25% in adenomas) or Akino *et al.*'s study (42% in CRCs and 43% in adenomas). However, the methylation frequency of RASSF2 in colorectal neoplasia was much higher than that of RASSF1 even in our results. Thus, the methylation of RASSF2, but not that of RASSF1, should have a distinct function during CRC development.

There were differences between adenomas and cancers in the locational distribution of neoplasm with genetic and/or epigenetic alterations of the Ras signalling pathway, although similar trends were observed (Figure 2). The most striking difference was seen in the proximal colon. Some adenomas in the proximal colon carried neither *K-ras/BRAF* mutations nor RASSF2 methylation, while there were few cancers without these alterations. This suggested that disorders in the Ras signalling pathway can occur in the proximal colon neoplasia not only during the early period but also during the late period of cancer progression. Then we can propose three types of cancer development with respect to the Ras signalling pathway. The first type is, as frequently seen in the distal colon neoplasms and in some rectal neoplasms, a tumour with no Ras signalling pathway alterations. The next type is, as has been widely believed thus far, a tumour in which disorder of the Ras signalling pathway occurs during the early period of cancer development. This type was seen in a large proportion of rectal tumours as well as in some tumours in the proximal colon. The third type, observed mainly in the proximal colon as shown above, is a tumour in which disorder of the Ras signalling pathway occurs during the late period of cancer development. Thus, our results suggest that both the likelihood of involvement of Ras signalling disorder in CRC development and the time point when the disorder is likely to occur differ according to tumour location.

We found that RASSF2 was frequently methylated in serrated adenomas. Recent reports have shown that serrated adenomas have biological features distinct from other conventional adenomas or hyperplastic polyps. In particular, these tumours are characterised by a high frequency of carrying *BRAF* mutation and a high frequency of CpG island methylation (CpG island methylator phenotype-high) (Park *et al*, 2003; Kambara *et al*, 2004; O’Brien *et al*, 2004), and are considered precursor lesions of CRC with microsatellite instability. Consistent with previous reports, in our study, *BRAF* mutation was frequently observed in serrated adenomas (5 out of 8, 63%). In addition, most of serrated adenomas carried RASSF2 methylation (6 out of 8, 75%). Consequently, serrated adenomas were likely to have both *K-ras/BRAF* mutations and RASSF2 methylation (5 out of 8, 63%), despite relatively dispersed locational distribution (Figure 2). As previously reported, methylation of RASSF2 in those tumours may be affected by CpG island methylator phenotype status (Minoo *et al*, 2006). Alternatively, however, a specific synergistic correlation between *BRAF* mutation and RASSF2 methylation may function in Ras signalling disorders during the progression of serrated adenomas.

There are limitations to our study. In particular, selection bias of collected tumours may inevitably exist even though tumours were collected consecutively. Because our institute is a tertiary care gastroenterology facility, patients with tumours that could not be easily treated in other hospitals were likely to be referred to our hospital. As a result, our collected series may be composed of uncommon fractions of colorectal neoplasm. In fact, the proportions of flat-type adenomas and rectal carcinomas were relatively high in our study. The relatively small number of adenoma samples as well as that of cancer samples would also be a limitation.

In conclusion, we demonstrated that RASSF2 methylation is of importance as well as *K-ras/BRAF* mutations during the progression of colorectal tumours. In addition, these genetic and epigenetic alterations in the Ras signalling pathway are likely to function synergistically. More importantly, however, both the likelihood and time point of the occurrence of these alteration differ according to tumour location. These results suggest that disorders in the Ras signalling pathway are not uniformly involved in the development of CRC. Frequency and the time point of the occurrence of Ras signalling disorders differ according to colorectal neoplasia’s characteristics, particularly the location.

## Figures and Tables

**Table 1:**
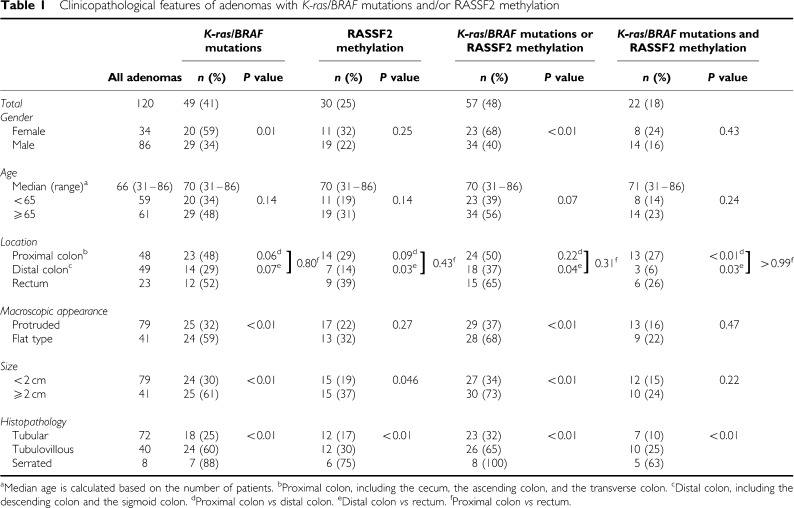
Clinicopathological features of adenomas with *K-ras/BRAF* mutations and/or RASSF2 methylation

**Table 2:** Univariate and multivariate analyses of factors for adenomas carrying both *K-ras/BRAF* mutations and RASSF2 methylation

		**Both *K-ras/BRAF* mutation and RASSF2 methylation**		
	**All adenomas**	***n* (%)**	**Univariate analysis OR (95% CI)**	**Multivariate analysis OR (95% CI)**
Total	120	21 (18)		
Female	34	8 (24)	1.73 (0.64–4.64)	1.51 (0.50–4.53)
Age (⩾65)	61	14 (23)	2.21 (0.82–5.95)	1.56 (0.54–4.53)
Distal colon	49	3 (6)	0.19 (0.05–0.69)^†^	0.13 (0.03–0.58)^‡^
Flat type	41	9 (22)	1.43 (0.55–3.69)	0.54 (0.16–1.84)
Size (⩾2 cm)	41	10 (24)	1.99 (0.77–5.19)	1.86 (0.59–5.94)
Serrated adenoma	8	5 (63)	10.00 (2.17–46.00)^*^	11.11 (1.96–63.00)^**^

Abbreviations: OR, odds ratio; CI, confidence interval. ^†^*P*=0.01, ^‡^*P*=0.007, ^*^P=0.003, **P=0.007.
